# The Effect of Pharmacological and Non-Pharmacological Interventions on Headaches Induced by Intravenous Nitroglycerin Infusion: A Systematic Review

**DOI:** 10.34172/jcvtr.33617

**Published:** 2026-06-30

**Authors:** Fatemeh Ahmadi, Masoumeh Bagheri Nesami, Mobina Abedinpour

**Affiliations:** ^1^Student Research Committee, Mazandaran University of Medical Sciences, Sari, Iran; ^2^Traditional and Complementary Medicine Research Center, Addiction Institute, Mazandaran University of Medical Sciences, Sari, Iran

**Keywords:** Infusion, Nitroglycerin, Headache, Intervention, Pharmacological, Non-Pharmacological

## Abstract

Nitroglycerin is a rapid-acting vasodilator commonly used as a first-line agent for the treatment of angina. Some patients receiving intravenous nitroglycerin infusion experience headaches as a side effect. Pain management is a critical priority for these patients. Therefore, this study aimed to determine the effect of pharmacological and non-pharmacological interventions on headaches induced by nitroglycerin infusion. This systematic review was conducted by searching the databases of SID, Magiran, Irandoc, Civilica, Science Direct, Web of Science, PubMed, Scopus, ProQuest, World Health Organization (WHO) and US Food and Drug Administration (FDA) and Google Scholar using the keywords "Nitroglycerin", "Glyceryl trinitrate", "Headache", "Cephalgia", "Cephalodynia", "Interventions" and "Infusion". After removing duplicates, studies were first screened based on inclusion and exclusion criteria, and their quality was then assessed. Finally, a total of 13 studies were entered into the systematic review. The total number of participants in all studies included in the review was 566 individuals aged 18 years and older. Eight studies (61.5%) addressed pharmacological interventions, and five studies (38.5%) addressed non-pharmacological interventions. The review of studies indicated that the use of cold compresses on the forehead, lavender essential oil aromatherapy, foot reflexology, sodium valproate, sumatriptan, and prednisone have proven to be effective treatments for headaches induced by nitroglycerin infusion; their use is recommended in the order mentioned for alleviating nitroglycerin-induced headaches. The use of cold compresses on the forehead, lavender essential oil aromatherapy, foot reflexology, sodium valproate, sumatriptan, and prednisone positively impacted the reduction of headaches induced by nitroglycerin infusion.

## Introduction

 Cardiovascular diseases (CVDs) rank as the primary cause of death worldwide, leading to a considerable number of fatalities and disabilities. In 2021, CVDs were linked to 20.5 million deaths, making up about one-third of all deaths globally.^1^ Nitroglycerin (NTG) is a rapid-acting vasodilator commonly used in emergency departments as a first-line agent for the treatment of angina and the management of chest pain associated with acute coronary syndromes. This drug is also used as a therapeutic option for acute heart failure, pulmonary edema, and aortic dissection.^2^ The most common route of NTG administration in intensive care units and emergency departments is intravenous infusion.^3^ A study has shown that early initiation of NTG infusion is associated with prolonged survival in patients with acute heart failure.^4^ However, studies have demonstrated that the vasodilatory effects of nitrates can lead to side effects in patients.^5,6^ Nitroglycerin is converted into Nitric Oxide (NO) in the body.^2^ NO then activates the enzyme guanylyl cyclase, which converts guanosine triphosphate (GTP) to guanosine 3’,5’-monophosphate (cGMP) in vascular smooth muscle and other tissues. cGMP activates several protein kinase-dependent phosphorylation that increase calcium reabsorption into the sarcoplasmic reticulum, increase extracellular calcium, and open calcium-gated potassium channels.^7^ This ultimately leads to dephosphorylation of myosin light chains in smooth muscle fibers. This activity causes relaxation of smooth muscle within blood vessels, thereby producing the desired vasodilator effect.^8^ At the same time, side effects such as headaches may occur, which are due to the sudden dilation of cerebral vessels and changes in intracranial pressure.^3^

 NTG-induced headache is the most common side effect of nitrate therapy^9^, occurring in 19-75% of patients depending on the dose of the drug.^10^ This side effect limits the use of the drug in some patients^11^, with nearly 10% unable to continue nitrate therapy due to intolerable headaches.^12^ Nitrate-induced headaches typically manifest in one of two ways: “immediate” headaches, which are mild to moderate in intensity and occur within one hour after drug administration, and “delayed” headaches, which occur 3 to 6 hours after nitrate use and are much more severe, often accompanied by migraine-like symptoms. Delayed migraines appear to be dose-dependent and more likely to occur in individuals with a family history of migraines.^12^ Additionally, a study has shown that NTG produces a type of headache with characteristics and vascular manifestations similar to migraine in both healthy individuals and migraineurs, although it is reversible and treatable under controlled conditions.^13^

 Similar to many other conditions, the treatment of NTG-induced headaches is approached through both pharmacological and non-pharmacological methods.^14^ Currently, pharmacological treatments are more commonly used.^15^ The alleviation of NTG-induced headaches has been tested using migraine treatments, some of which have been effective, while others have not.^13^ Previous studies have shown that the administration of valproate^16^, prednisone^17^, sumatriptan^18-20^, aromatherapy with rose^11^ or lavender^11,14,21,22^, and intranasal cocaine and lidocaine^23^ is effective in reducing the intensity of NTG-induced headaches. In contrast, propranolol^24^, mirtazapine^25^, olcegepant^26^, and tonerbasat^27^ have not been effective in relieving NTG-induced headaches. Additionally, the use of acetylcysteine has led to headache exacerbation.^28^

 Non-pharmacological methods are another approach used to manage pain. These methods work by increasing the sense of control, reducing feelings of helplessness, enhancing activity levels, decreasing stress and anxiety, lowering analgesic drug doses, and reducing pain intensity.^29^ In recent years, non-pharmacological methods have attracted the attention of patients and healthcare providers due to their lack of adverse effects and undesirable outcomes commonly associated with pharmacological interventions.^30^ There are numerous non-pharmacological approaches that can be used to reduce or eliminate pain.^31^ The effects of these methods have been explored in many studies, with results indicating that the use of cold compresses on the forehead at the start of NTG infusion^9^, foot reflexology^32^, and cold or heat therapy on the neck through a compress bag during migraine attacks^33^ are among the effective non-pharmacological interventions for reducing NTG-induced headaches.

 Therefore, given the abundance of studies in this field and the lack of a comprehensive synthesis of this information, and considering that NTG-induced headache is a significant issue for patients with no definitive treatment to alleviate their pain completely, this study was conducted to determine the effect of pharmacological and non-pharmacological interventions on NTG-induced headaches. The aim is to assist in alleviating these patients’ pain and discomfort and offer basic information for nurses and physicians in managing NTG-induced headaches.

## Materials and Methods

 This systematic review was conducted in 2025. The study has been registered on PROSPERO (CRD420251130272).

###  Search strategy

 International databases—including Web of Science, Medline via PubMed, Scopus, ScienceDirect, and ProQuest, Iranian databases—including the Scientific Information Database (SID) and Magiran—and the Google Scholar search engine, World Health Organization (WHO) and US Food and Drug Administration (FDA) were independently searched by two reviewers (BNM and AF) from the inception of these databases until 7 September 2025. Additionally, the IRANDOC and Civilica databases were examined for relevant theses and conference proceedings. The reference lists of the retrieved articles were also checked for additional sources. The keywords “Nitroglycerin”, “Glyceryl trinitrate”, “Headache”, “Cephalgia”, “Cephalodynia”, “Interventions” and “Infusion” were searched in the above-mentioned databases using the Boolean operators AND and OR. The search strategies are presented in Table 1.

**Table 1:** Search strategies

**Database**	**Search Strategy**	**Filters**
PubMed/Medline	(((Nitroglycerin [Title/Abstract]) OR (Glyceryl trinitrate [Title/Abstract])) AND ((Infusion [Title/Abstract]) OR (Intravenous Infusion [Title/Abstract]))) AND (((Headache [Title/Abstract]) OR (Cephalgia [Title/Abstract])) OR (Cephalodynia [Title/Abstract]))	Article Language: English, Persian
Scopus	(TITLE-ABS-KEY (Nitroglycerin) AND TITLE-ABS-KEY (Glyceryl AND Trinitrate) AND TITLE-ABS-KEY (Infusion) OR TITLE-ABS-KEY (Intravenous AND Infusion) AND TITLE-ABS-KEY (Headache) OR TITLE-ABS-KEY (Cephalgia) OR TITLE-ABS-KEY (Cephalodynia))	Subject areas: Medicine Document type: Article Source type: Journal
Web of Science	(((TS = (Nitroglycerin) OR TS = (Glyceryl trinitrate)) AND (TS = (Infusion) OR TS = (Intravenous infusion))) AND (TS = (Headache) OR TS = (Cephalgia) OR TS = (Cephalodynia)))	Document type: Article
Science Direct	((Nitroglycerin) OR (Glyceryl trinitrate)) AND ((Infusion) OR (Intravenous infusion)) AND ((Headache) OR (Cephalgia) OR (Cephalodynia))	Article type: Research article
ProQuest	ti(Nitroglycerin) OR ti(Glyceryl trinitrate) AND ti(Infusion) AND ti(Headache) OR ti(Cephalalgia) OR ti(Cephalodynia)	No search filters were applied
Google Scholar	“Nitroglycerin” OR “Glyceryl trinitrate” AND “Infusion” AND “Headache” OR “Cephalalgia” OR “Cephalodynia”	Year of publication: Anytime Key words anywhere in the article Sort by relevance
SID	((Nitroglycerin) OR (Glyceryl trinitrate)) AND ((Infusion) OR (Intravenous infusion)) AND ((Headache) OR (Cephalgia) OR (Cephalodynia))	No search filters were applied
Magiran	((Nitroglycerin) OR (Glyceryl trinitrate)) AND ((Infusion) OR (Intravenous infusion)) AND ((Headache) OR (Cephalgia) OR (Cephalodynia))	No search filters were applied

###  Inclusion & exclusion criteria

 Studies that met the following criteria were included in the review: (a) interventional studies on the effects of pharmacological (chemical or herbal) or non-pharmacological (complementary medicine) interventions on NTG-induced headaches, and (b) studies published in Persian or English. Two articles that were not fully accessible and were only available as abstracts presented at conferences were excluded from the review due to insufficient information. Furthermore, no related theses were found in the ProQuest and IRANDOC databases. Duplicate articles were removed using EndNote software.

###  Selection process

 Two reviewers (BNM and AF) independently reviewed the titles and abstracts of the articles. Articles were included or excluded from the study based on predetermined criteria. During the initial screening, the selection process involved evaluating the relevance of the titles to the study objectives, assessing the abstracts for alignment with the study objectives, and evaluating the full text of the articles for relevance to the study objectives. Disagreements between reviewers were resolved by further discussion or, if necessary, by the participation of a third reviewer (AM). The flowchart depicting the selection process of the articles is shown in Figure 1.

**Figure 1:**
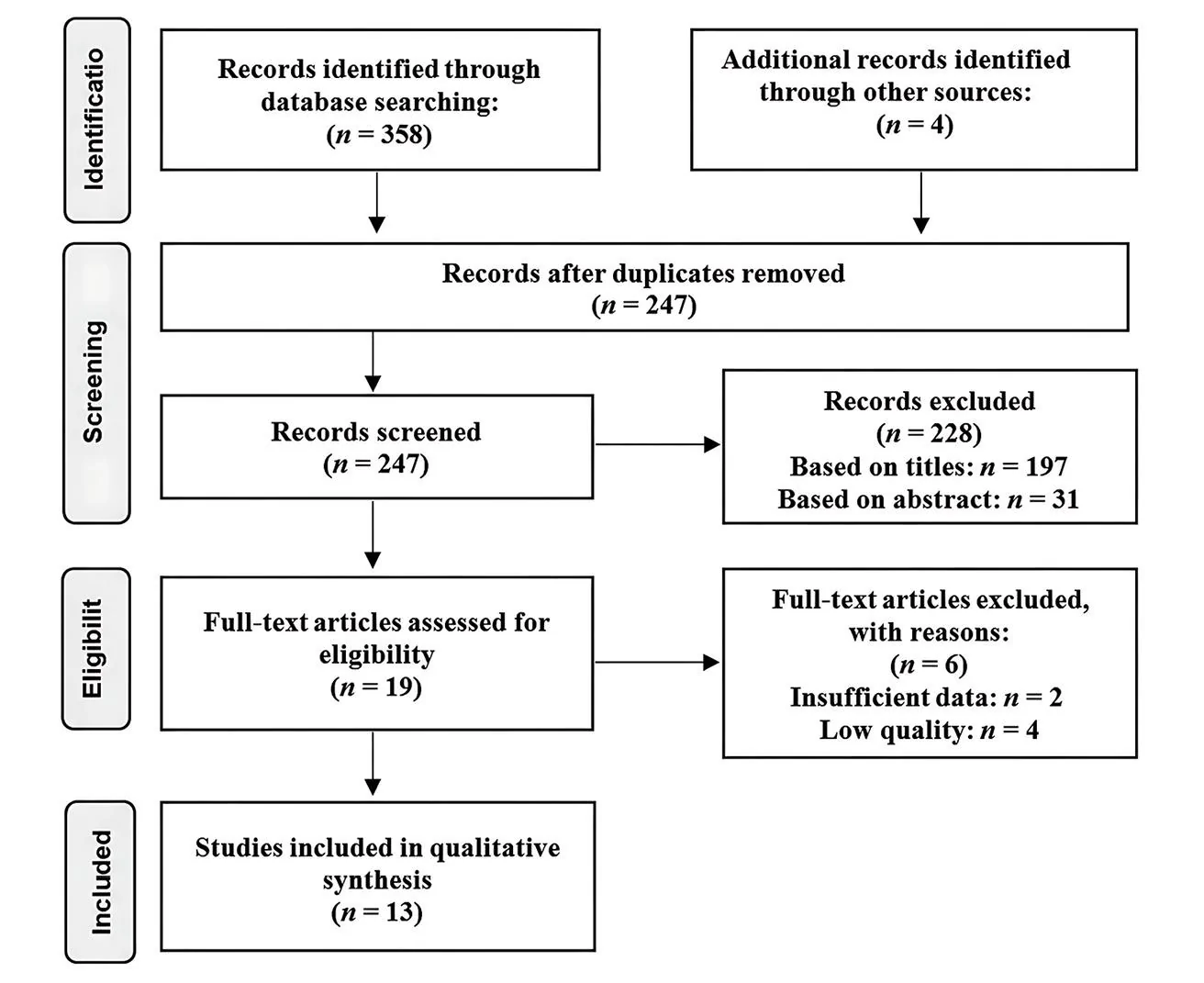
Flowchart of the Selection process

###  Risk-of-bias and quality assessment

 Articles were assessed separately by two reviewers (BNM and AF) using the Cochrane Risk of Bias Tool for Randomized Trials (ROB 2).^34^ The Rob 2 tool includes Five domains: randomization process, deviation from intended interventions, missing outcome data, measurement of outcome, selection of the reported result. Assessors’ responses were categorized as yes, probably yes, probably no, no and no information (Figures 2 and 3). Disagreements were resolved by joint reassessment of the original articles with a third reviewer (AM). The methodological quality of the articles was also assessed by two independent investigators (BNM and AF) using the standard JADAD checklist. The JADAD checklist includes three criteria: reporting of randomization, blinding, and study withdrawal. Studies that received scores of 1 to 2 and 3 to 5 were classified as low and high quality, respectively.^35^ Disagreements between reviewers were resolved by further discussion or, if necessary, by the participation of a third reviewer (AM). After quality assessment, four studies^11,18,21,33^ were excluded because they received a score of less than 3, and finally, twelve studies were included in the qualitative synthesis. The quality assessment scores of the included studies are presented in Table 2.

**Figure 2:**
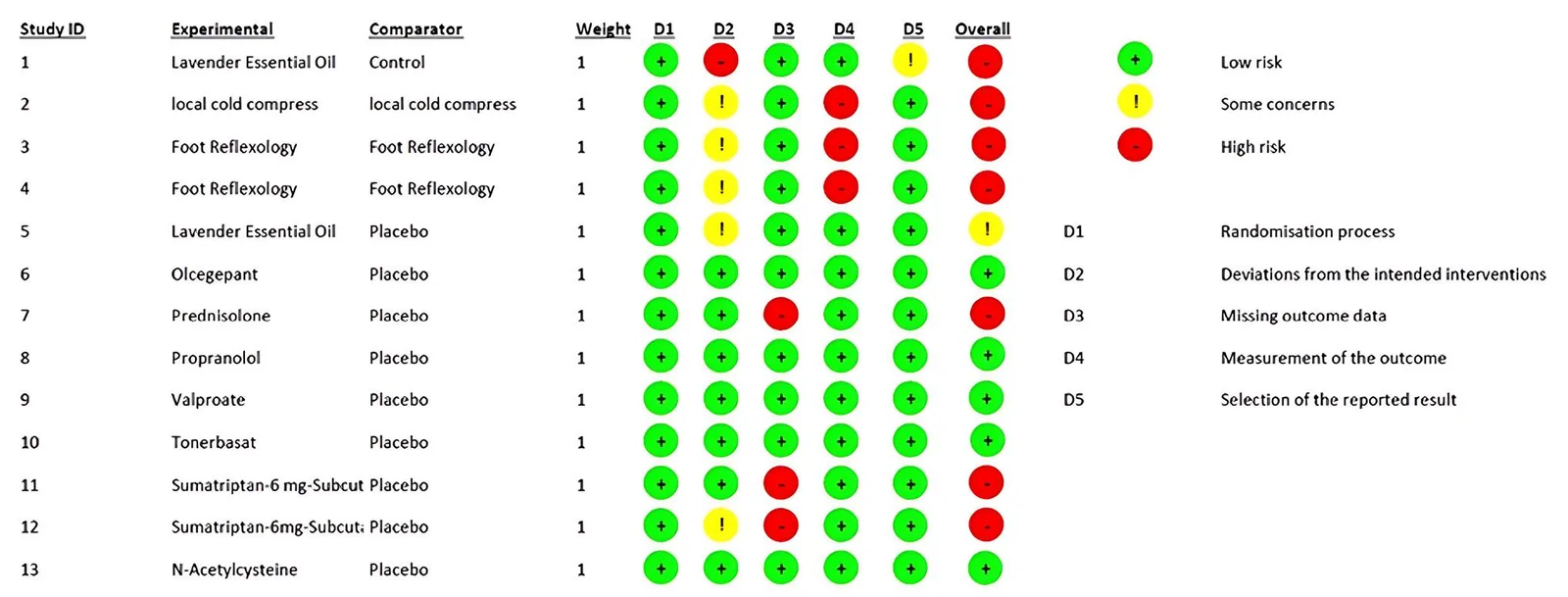
Risk of bias chart for randomized controlled trials using the Excel tool for implementing Rob 2

**Figure 3:**
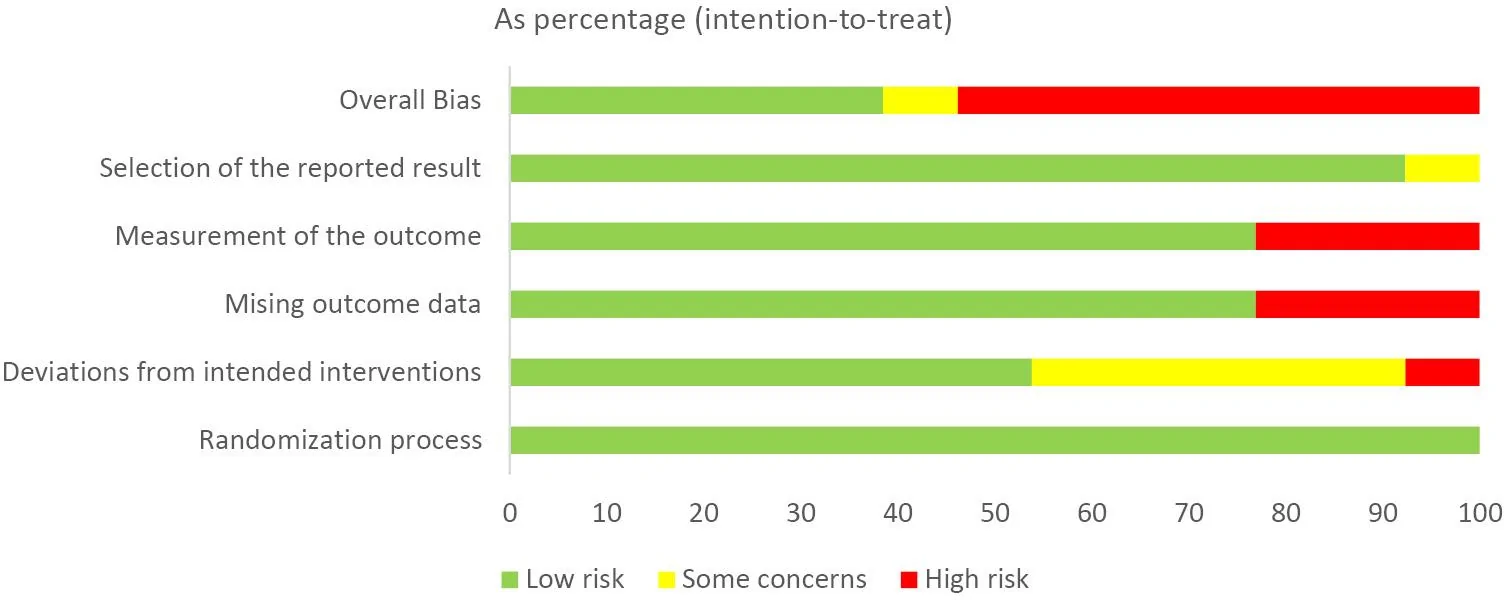
Risk of bias summary for randomized controlled trials using Excel tool to implement Rob 2

**Table 2:** Quality assessment of the included articles using the standard JADAD checklist

**No.**	**Article**	**Randomization**		**Blinding**		**withdrawals or dropouts**	**Overall Score**
		**Random Allocation**	**Randomization Method**	**Blinding**	**Blinding Method**		
1	Ezati-Soleiman et al^22^	1	1	0	0	1	3
2	Uğurlu & Enc^9^	1	1	1	1	1	5
3	Fatai et al^32^	1	1	1	1	1	5
4	Imani et al^36^	1	1	1	1	1	5
5	Zarifnejad et al^14^	1	1	0	0	1	3
6	Tvedsko et al^26^	1	0	1	1	1	4
7	Tfelt‐Hansen et al^17^	1	0	1	1	0	3
8	Tvedskov et al^24^	1	0	1	1	1	4
9	Tvedskov et al^16^	1	0	1	1	1	4
10	Tvedskov, Iversen & Olesen^27^	1	0	1	1	1	4
11	Schmetterer et al^20^	1	0	1	1	0	3
12	Iversen & Olesen^19^	1	0	1	1	0	3
13	Iversen^28^	1	0	1	1	1	4

###  Data extraction

 After extracting the required information, the results were first summarized in a data extraction table and then analyzed manually. The extracted data were organized according to variables including author, year of publication, title, study type, sample size, inclusion and exclusion criteria, drug/dose, intervention duration, pain assessment tool, mean pain score, results, conclusion, and difference in percentage/mean/median between the two groups (intervention and control/placebo) at the final measurement (Table 3 and 4).

**Table 3:** Non-pharmacological Intervention

**Study ID**	**Authors & Year of Publication**	**Study Design**	**Sample Size & Groups**	**Intervention**	**Duration of Intervention**	**Tools**	**Results & Conclusion**	**Difference in Percentage/ Mean/Median Between Groups at the Final Measurement**
1	Ezati-Soleiman et al^22^	randomized clinical trial	Sample Size: n = 90 Lavender Essential Oil Group: n = 30 Placebo Group: n = 30 Acetaminophen Group: n = 30	Inhalation of Lavender (Three Drops)	Lavender Group: Inhalation of three drops of lavender essential oil on a cotton gauze 15 minutes before nitroglycerin IV infusion Control Group: routine care for headaches caused by nitroglycerin. This involved the administration of 500 mg of acetaminophen following the nitroglycerin infusion whenever the patient reported experiencing a headache. Acetaminophen Group: 500 mg of oral acetaminophen about 15 minutes prior to starting the IV nitroglycerin infusion.	Visual Analog Scale (VAS)	At the 60-minute mark, the average headache intensity was 1.37 ± 1.10 in the lavender group and 2.50 ± 2.43 in the control group, whereas no headaches were reported in the acetaminophen group. Headache severity showed significant variation throughout the intervention minutes among the three groups (*P* < 0.001).	Difference in the median headache scores between the lavender and control groups 60 minutes after the intervention: 1.63
2	Uğurlu & Enc^9^	Pretest-Posttest Without Control Group	Sample Size: n = 70 Group 1: n = 35 Group 2: n = 35	Cold Compress (Forehead)	Group 1: Application of a local cold compress for the first 20 minutes of NTG infusion Group 2: Application of a local cold compress for 20 minutes in patients experiencing a headache after NTG infusion (no cold compress applied at the start of the infusion)	Visual Analog Scale (VAS)	Patients in the intervention group had significantly lower VAS scores before (5.75) and after (2.00) using the the cold compress (*P* = 0.000). The application of a local cold compress at the start of NTG infusion has a pain prophylactic role and reduces headache intensity in patients experiencing NTG-induced headaches.	Difference in headache occurrence percentage between the groups after 12 hours: 27.5%
3	Fatai et al^32^	Randomized Clinical Trial	Sample Size: n = 60 Intervention Group: n = 30 Control Group: n = 30	Foot Reflexology using the Ingham method on both feet. Control Group: Superficial Foot Touch	intervention lasted 10 minutes (5 minutes per foot) and was followed by a second 10-minute reflexology intervention after a 15-minute interval.	McGill Pain Questionnaire Short-Form (MPQ-SF)	There was a significant difference after the intervention between the two groups (*P* < 0.001). Foot reflexology is a feasible intervention for reducing headaches in patients receiving NTG infusion and is associated with headache improvement.	Difference in mean headache scores between the two groups after the second intervention (after 35 minutes): 5.43
4	Imani et al^36^	Randomized Clinical Trial	Sample Size: n = 75 Intervention Group: n = 25 Placebo Group: n = 25 Control Group: n = 25	Foot Reflexology using the Ingham method on both feet with the thumb of the right hand on the brain reflex points Placebo Group: an unrelated point on the heel (not associated with the brain) control group: did not receive any massage	intervention lasted 20 minutes (10 minutes per foot) and was followed by a second 20-minute reflexology intervention after a 3 hours interval.	Numerical Rating Scale (NRS)	There was a significant difference among the three groups post-intervention (*P* = 0.000). Reflexology massage can reduce the intensity of NTG-induced headaches	Pairwise comparisons of differences in the mean headache score between the three groups after the second intervention (after 3 hours and 40 minutes): Reflexology vs. Placebo: 3.4 Intervention vs. Control: 4.2 Placebo vs. Control: 0.8
5	Zarifnejad et al^14^	Clinical Trial	Sample Size: n = 135 Lavender Essential Oil Group: n = 45 Placebo Group: n = 45 Acetaminophen Group: n = 45	Inhalation of Lavender (Three Drops)	Lavender/placebo Group: Inhalation of three drops of lavender essential oil/ placebo on a cotton ball for 30 minutes Acetaminophen Group: Administration of a single dose of 325 mg acetaminophen tablet	Visual Analog Scale (VAS)	Pairwise comparisons revealed a significant reduction in headache in the lavender group compared to the acetaminophen group (*P* = 0.001) and in the acetaminophen group compared to the placebo group (*P* = 0.001). Aromatherapy with lavender essential oil may effectively reduce headaches in patients receiving NTG infusion.	Difference in the median headache scores between the lavender and placebo groups 60 minutes after the intervention: 4.4

**Table 4:** Pharmacological Intervention

**Study ID**	**Authors & Year of Publication**	**Study Design**	**Sample Size & Groups**	**Drug & Dose**	**Duration of Intervention**	**Tools**	**Results & Conclusion**	**Difference in Percentage/ Mean/Median Between Groups at the Final Measurement**
6	Tvedsko et al^26^	Double-Blind, Randomized, Placebo-Controlled, Crossover Study	Sample Size: n = 13	10 mg Olcegepant or Placebo (5% Xylitol)	Day 1: Initial infusion of 0.5 mg/kg/min NTG for 20 minutes, followed by a 10 mg infusion of Olcegepant 40 minutes later over 10 minutes Day 2: Initial infusion of 0.5 mg/kg/min NTG for 20 minutes, followed by a placebo infusion (5% xylitol) for 10 minutes	The 0-to-10 Verbal NRS	Peak headache scores ranged from 0 to 10 (median of 5) in patients receiving NTG plus placebo and from 0 to 9 (median of 4) in those receiving NTG plus Olcegepant (*P* = 0.69). Olcegepant, a CGRP receptor antagonist (BIBN4096BS), does not have a preventive effect on NTG-induced migraines.	Difference in the median peak headache scores between the intervention and placebo groups after 12 hours: 1
7	Tfelt‐Hansen et al^17^	Double-Blind, Randomized, Placebo-Controlled, Crossover Study	Intervention Group: n = 15 Placebo Group: n = 15	150 mg Prednisolone	Intervention Group: Administration of prednisolone at 12-hour intervals prior to NTG infusion (0.5 µg/kg/min for 20 minutes). Placebo Group: Administration of placebo Washout Period: 3 weeks	The 0-to-10 Verbal NRS	Findings showed significantly lower peak headache scores over 12 hours after the use of prednisolone (*P* < 0.01), but peak headache and headache response did not significantly change (*P* < 0.07). Pretreatment with prednisolone did not reduce immediate NTG-induced headaches and did not prevent delayed headache frequency, but significantly reduced the severity of delayed NTG-induced headaches.	Difference in the mean peak headache scores between the two groups after 12 hours: 2
8	Tvedskov et al^24^	Double-Blind, Randomized, Placebo-Controlled, Multicenter Crossover Study	Sample Size: n = 35 (19 Patients with Migraine, 16 Controls)	160 mg Propranolol or Placebo	Intervention Group: Administration of 160 mg propranolol daily for 13 days prior to the date of NTG infusion Placebo Group: Administration of placebo daily for 13 days prior to the date of NTG infusion Washout Period: 14 days	The 0-to-10 Verbal NRS	NTG-induced headaches were more pronounced in migraine patients compared to healthy individuals, both with (*P* = 0.003) and without (*P* = 0.017) pretreatment with propranolol. Propranolol was not effective in treating NTG-induced headaches and migraines.	Difference in the median peak headache scores between the two groups after 12 hours: 1
9	Tvedskov et al^16^	Randomized, Double-Blind, Crossover Study	Sample Size: n = 12 Intervention Group: n = 6 Placebo Group: n = 6	1000 mg Sodium Valproate or Placebo Tablets	Intervention Group: Administration of sodium valproate for at least 13 days Placebo Group: Administration of placebo for at least 13 days Washout Period: 14 days at least	The 0-to-10 Verbal NRS	The mean peak headache intensity was 1 (range of 0–9) after the use of valproate compared to 4.5 (range of 0–8) after the use of placebo (*P* = 0.12). Valproate was effective in managing NTG-induced headaches and migraines.	Difference in the median headache intensity scores between the two groups after 12 hours: 3.5
10	Tvedskov, Iversen& Olesen^27^	Randomized, Double-Blind, Crossover Study	Sample Size: n = 15 Day 1: n = 9 Day 2: n = 9	40 mg Oral Dose of SB-220453 (Tonerbasat) or Placebo Tablets	Intervention Group: Administration of a Tonerbasat tablet before Intravenous infusion NTG Placebo Group: Administration of a placebo tablet Washout Period: 11 days at least	The 0-to-10 Verbal NRS	No significant reduction was observed in headache scores. There was no decrease in the number of individuals with delayed headaches or migraine attacks.	Difference in the mean headache intensity scores between the two groups after 12 hours: 1.2
11	Schmetterer et al^20^	Double-Blind, Randomized, Placebo-Controlled, Crossover Study	Sample Size: n = 10 (Healthy Male Participants)	Subcutaneous Injection of 6 mg Sumatriptan (0.5 mL) or Placebo (0.5 mL Physiological Saline Solution)	Intervention Group: Subcutaneous administration of sumatriptan Placebo Group: Subcutaneous administration of placebo Washout Period: 2-6 days	Participants were asked twice whether they experienced pain or discomfort (20 minutes after the administration of sumatriptan or placebo and 10 minutes after the start of NTG infusion).	Following the NTG infusion, the velocity of the middle cerebral blood flow decreased by 13.3% from baseline after placebo treatment, compared to 2.2% after the consumption of sumatriptan (treatment effect, + 10.8%; *P* < 0.05).	Not reported
12	Iversen & Olesen^19^	Randomized, Double-Blind Crossover Study	Sample Size: n = 10	Subcutaneous Injection of 6 mg Sumatriptan or Placebo (Isotonic Saline)	Intervention Group: Subcutaneous administration of 6 mg sumatriptan in the deltoid region Placebo Group: Subcutaneous administration of placebo (isotonic saline) in the deltoid region	The 0-to-10 Verbal NRS	Sumatriptan reduced NTG-induced headache (mean score of 1.5 vs. 4 after placebo; *P* < 0.01). Whether sumatriptan acts on preventive receptors cannot be determined based on the results of this study	Difference in the median headache scores at the time of NTG infusion between the two groups: 2.5
13	Iversen^28^	Randomized, Placebo-Controlled, Crossover Study	Sample Size: n = 11	100 mg/kg N- Acetylcysteine diluted in 100 mL isotonic dextrose solution or Placebo (Saline Solution)	Day 1: Intravenous injection of N-acetylcysteine over 15 minutes, followed by an infusion of NTG for 20 minutes Day 2: Intravenous injection of saline over 15 minutes, followed by an infusion of NTG for 20 minutes Washout Period: 6 days at least	The 0-to-10 Verbal NRS	Headaches during both NTG infusion sessions lasted longer after the administration of N-acetylcysteine compared to after that of placebo.	Difference in the median peak headache scores between the intervention and placebo groups after 3 hours: 2

## Results

 The results indicated either a positive effect or no effect of pharmacological (chemical and herbal) and non-pharmacological (complementary medicine) interventions on reducing headaches induced by NTG infusion. The total number of participants across all the studies included in the systematic review (*n* = 13) was 566. It should be also noted that all of the participants were aged 18 and older. The pain assessment tools used in the studies included the Visual Analog Scale (VAS), the Short-Form McGill Pain Questionnaire (SF-MPQ), and the Numerical Rating Scale (NRS). A summary of the details of the included studies is presented in Table 3 and 4.

## Discussion

 The aim of this study was to evaluate the effects of pharmacological (chemical and herbal) and non-pharmacological (complementary medicine) interventions on headaches induced by NTG infusion. To this end, nine studies on pharmacological interventions^14,16,17,19,20,24,26-28^ and Five on non-pharmacological ones^9,14,22,32,36^ were examined. Among these, eight studies were on preventive treatments administered before NTG infusion.^9,16,17,19,20,22,24,27,28^

 Uğurlu and Enc investigated the effect of local cold compresses applied to the forehead in two groups of patients experiencing NTG-induced headaches. The first group (35 patients) received local cold compresses during the first 20 minutes of NTG infusion, while the second group (35 patients) received compresses for 20 minutes after the onset of headache. The results indicated that applying local cold compresses at the beginning of NTG infusion prevented the headache, whereas using them after the onset of NTG-induced headache reduced its intensity. The incidence of NTG-induced headache was 25.8% in the group that received cold compresses during the first 20 minutes of infusion compared to 53.3% in the group without compress application. Accordingly, a 27.5% difference was shown between the two groups in this regard.^9^

 Fatai et al conducted a controlled clinical trial to assess the effect of foot reflexology on NTG-induced headaches. Participants were randomly assigned to two groups of 30 individuals. In the intervention group, reflexology was performed for 10 minutes (5 minutes per foot) and followed by a second 10-minute reflexology session after a 15-minute interval. The SF-MPQ with a maximum score of 60 was used to measure headache severity. The results showed a significant difference in headache intensity with a difference of 5.43 in the mean score of NTG-induced headache between the control and intervention groups after the intervention. After the intervention, the mean score of NTG-induced headaches was shown to be 13.63 and 8.20 in the control and intervention groups, respectively.^32^ This difference translates to 0.91 when adjusted to a 10-point scale, which may appear small due to the limited sample size. Imani et al also conducted a clinical trial to evaluate the effect of foot reflexology on NTG-induced headaches in 75 patients. Participants were randomly assigned to three groups (intervention, placebo, and control), each consisting of 25 individuals. Reflexology was performed on the big toe for 20 minutes (10 minutes per foot) in the intervention group, while the placebo group received the same intervention on the heel. A second reflexology session was conducted three hours after the first. A 10-point NRS was used to assess headache severity. The difference in the mean headache scores between the intervention and control groups was reported as 4.2 after 3 hours and 40 minutes.^36^ These findings suggest that both the duration of reflexology and the interval between sessions play a significant role in alleviating NTG-induced headaches. Based on a comparison between the results of the studies by Fatai et al and Imani et al, it appears that performing reflexology for 20 minutes (10 minutes per foot) per session and allowing a three-hour interval between sessions is more effective in reducing NTG-induced headaches than shorter sessions (10 minutes total, 5 minutes per foot) with a 15-minute interval between interventions.

 Ezzati-Soleman et al conducted a clinical trial to assess the impact of lavender aromatherapy and acetaminophen used prophylactically on comfort levels and headaches induced by nitroglycerin in patients with acute coronary syndrome. Patients were randomly assigned to three groups of lavender essential oil, acetaminophen, and control (30 subjects per group). Individuals in the lavender group inhaled three drops of 2% lavender essential oil on a cotton swab 15 minutes before the intravenous administration of nitroglycerin. The acetaminophen group was given 500 mg of acetaminophen orally about 15 minutes before the nitroglycerin infusion began. Those in the control group received standard care for headaches caused by nitroglycerin, which involved administering 500 mg of acetaminophen after nitroglycerin was injected at the time they reported a headache. Headache severity was measured using a VAS at 5, 10, 15, and 60 minutes after the start of the nitroglycerin infusion. The results of their study showed significant differences in headache severity between the three groups. The difference in mean headache scores between the lavender and control groups 60 minutes after the intervention was 1.63.^22^ Zarifnejad et al also conducted a clinical trial to evaluate the effect of aromatherapy with lavender essential oil on NTG-induced headaches. Patients were randomly assigned to three groups of lavender essential oil, acetaminophen, and placebo (*n* = 45 in each group). Participants in the lavender group inhaled three drops of 2% lavender essential oil placed on a cotton ball for 30 minutes after complaining of a headache, while those in the placebo group inhaled three drops of liquid paraffin under similar conditions. The acetaminophen group received a single 325 mg acetaminophen tablet. Headache severity was measured using the VAS before and 15, 30, and 60 minutes after the intervention. The results of their study revealed significant differences in headache severity among the three groups. Pairwise comparisons indicated a statistically significant reduction in headache severity in the lavender group compared to the acetaminophen group and in the acetaminophen group compared to the placebo group. The difference in median headache scores between the lavender and placebo groups 60 minutes after the intervention was reported as 4.4.^14^ When comparing these findings with previous studies that examined the effects of aromatherapy with 2% lavender 15 minutes before intravenous nitroglycerin infusion^22^ and foot reflexology on NTG-induced headaches^32,36^, it appears that aromatherapy with 2% lavender for 30 minutes after a headache complaint is more effective in reducing headache severity than foot reflexology.

 Moradi et al also reported that aromatherapy with lavender essential oil effectively alleviates NTG-induced headaches. The incidence of headache 90 minutes after the intervention was 25% in the lavender group compared to 50% in the control group, which indicates a 25% difference between the two groups.^11^ In the study by Uğurlu and Enc, the effect of local cold compresses on NTG-induced headaches was investigated. The percentage difference between the intervention and control groups 12 hours after the intervention was reported as 27.5%.^9^ This suggests that applying local cold compresses to the forehead may be more effective than lavender aromatherapy in managing NTG-induced headaches.

 Tvedskov et al conducted a double-blind, placebo-controlled, crossover study to investigate the effect of the CGRP receptor antagonist (Olcegepant) on NTG-induced headaches. On the first study day, patients received an initial NTG infusion at a dose of 0.5 µg/kg/min for 20 minutes. Forty minutes later, 10 mg of Olcegepant was infused over 10 minutes. On the second study day, patients underwent the same NTG infusion protocol, followed 40 minutes later by a placebo infusion (5% xylitol). Headache severity was assessed using the NRS. The results showed that Olcegepant was ineffective in preventing NTG-induced headaches. The difference in median peak headache intensity between the intervention and placebo groups after 12 hours was reported as 1.^26^

 Tfelt-Hansen et al conducted a double-blind, placebo-controlled, crossover study to evaluate the effect of prednisolone on NTG-induced headaches. Participants were randomly assigned to two groups of 15 individuals. The first group received a placebo, while the second group received 150 mg of prednisolone at 12-hour intervals before the NTG infusion (0.5 µg/kg/min for 20 minutes). The study included a 3-week washout period and headache severity was measured using the NRS. The findings indicated that prednisolone did not reduce immediate NTG-induced headaches, failed to prevent delayed headache occurrence, but significantly reduced the severity of delayed headaches caused by NTG. The difference in the mean peak headache intensity between the intervention and placebo groups after 12 hours was reported as 2.^17^ Based on a comparison between these findings with those of previous studies on the effects of local cold compresses^9^, aromatherapy with 2% lavender for 30 minutes after a headache complaint^14^, and foot reflexology^36^ on NTG-induced headaches, it can be concluded that prednisolone is less effective in alleviating NTG-induced headaches compared to these non-pharmacological interventions.

 Tvedskov et al conducted a randomized, double-blind, crossover study to evaluate the effect of propranolol on NTG-induced headaches. Participants were randomly assigned to two groups: one received 160 mg of propranolol daily and the other received a placebo for 13 days before the NTG infusion. The washout period lasted 14 days. Headache severity was measured using the NRS. It was indicated that propranolol was ineffective in preventing NTG-induced headaches and migraines. The difference in median peak headache intensity between the propranolol and placebo groups 12 hours after the intervention was reported as 1.^24^ In another crossover study, Tvedskov et al examined the preventive effect of valproate on NTG-induced migraine headaches. Patients were randomly assigned to two groups of six participants each. One group received 1000 mg of sodium valproate daily, while the other received a placebo for 13 days prior to the NTG infusion (0.25 mg/kg/min for 20 minutes). The washout period was at least 14 days. Headache severity was assessed using the NRS. The results showed that sodium valproate was effective in reducing NTG-induced headaches and migraines. The difference in median headache severity scores between the intervention and placebo groups was reported as 3.5 after 12 hours.^16^ When comparing these findings with those of previous studies on interventions for NTG-induced headaches, including local cold compresses^9^, aromatherapy with 2% lavender for 30 minutes after a headache complaint^14^, foot reflexology^36^, and prednisolone^17^, it appears that sodium valproate has less impact compared to cold compresses, aromatherapy with 2% lavender for 30 minutes after a headache complaint, and reflexology but shows greater efficacy than prednisolone. However, it should be noted that the small sample size in the study on the effect of valproate might have influenced the reported effect size.

 Tvedskov et al conducted a double-blind, crossover study to investigate the effect of Tonerbasat on NTG-induced headaches. Patients were randomly assigned to two groups of nine participants each. One group received a 40 mg Tonerbasat tablet, while the other group received a placebo. Both groups underwent intravenous NTG infusion (0.5 mg/kg/min for 20 minutes) 60 minutes after receiving Tonerbasat or placebo. The washout period was a minimum of 11 days. Headache severity was measured using the NRS. Based on the results, no significant reduction was found in immediate headache severity following the use of Tonerbasat (mean score of 3 versus 3.4). However, there was a tendency for reduced delayed headache severity in the Tonerbasat group (mean score of 3.8 versus 5). The difference in the mean headache severity between the intervention and control groups after 12 hours was reported to be 1.2.^27^

 Schmetterer et al conducted a double-blind, placebo-controlled, crossover study to assess the cerebrovascular and ocular hemodynamic effects of sumatriptan in a NTG headache model. Participants were randomly assigned to two groups: one received subcutaneous sumatriptan (6 mg in 0.5 mL), while the other received a placebo (0.5 mL physiological saline) prior to the NTG infusion (0.5 µg/kg/min for 20 minutes). Twenty minutes after sumatriptan or placebo administration and 10 minutes after the start of NTG infusion, participants were asked about their sensations or pain. The results showed that sumatriptan prevented NTG-induced vasodilation in the middle cerebral artery.^20^ Similarly, Fullerton et al demonstrated that subcutaneous or intravenous sumatriptan reduced NTG-induced headache and exerted a vasoconstrictive effect on the dilated middle cerebral artery caused by NTG infusion.^18^

 Based on the results of another study, it was found that subcutaneous administration of 6 mg sumatriptan significantly reduced NTG-induced headache compared to placebo (isotonic saline) with a difference of 2.5 in median headache scores during NTG infusion between the intervention and placebo groups.^19^ Based on comparison between these findings and those of previous studies on the effects of local cold compresses^9^, aromatherapy with 2% lavender for 30 minutes after a headache complaint^14^, foot reflexology^36^, and prednisolone^17^, it can be concluded that sumatriptan is more effective than prednisolone but less effective than cold compresses, aromatherapy with 2% lavender for 30 minutes after a headache complaint, foot reflexology, and sodium valproate in alleviating NTG-induced headaches.

 Iversen et al conducted a placebo-controlled crossover study to investigate the effect of acetylcysteine on NTG-induced headaches. Participants were randomly allocated to two groups. One group received 100 mg/kg of intravenous acetylcysteine, while the other received saline over a 15-minute period. Patients then underwent a NTG infusion with dose of 0.06 µg/kg/min for 20 minutes. A second NTG infusion was administered 120 minutes after the initial infusion. Headache severity was assessed using the NRS. Based on the findings, it was found that the headache duration during both NTG infusions was longer compared to the placebo after receiving acetylcysteine. The difference in median peak headache intensity scores between the intervention and placebo groups after 3 hours was reported as 2.^28^

 Limitations of this systematic review include heterogeneity in study designs, small sample sizes in some trials, and variation in outcome measures (VAS, NRS, SF-MPQ). These factors may influence the effect estimate and interpretation of results. Also, as meta-analysis was not performed, we were unable to quantitatively measure the cumulative effect, which is likely to be another limitation to the consistency of the evidence.

## Conclusion

 A comprehensive review of the published studies in both Persian and English revealed that various pharmacological and non-pharmacological strategies can be employed to alleviate NTG-induced headaches. The findings suggest that effective non-pharmacological approaches include the application of a cold compress to the forehead, aromatherapy using three drops of lavender essential oil 2% for 30 minutes after a headache complaint, and foot reflexology for 20 minutes (10 minutes per foot) followed by a second reflexology session three hours after the first. Among the pharmacological interventions, 1000 mg of sodium valproate, subcutaneous injection of 6 mg of sumatriptan, and 160 mg of prednisolone have shown efficacy in managing NTG-induced headaches. In contrast, other pharmacological agents such as propranolol, Olcegepant, Tonerbasat, and N-acetylcysteine did not demonstrate significant effectiveness, although differences in the timing of the final pain assessments across studies may have influenced these outcomes.

## Competing Interests

 The authors declare no conflict of interest.

## Ethical Approval

 The present study has been approved by the Ethics Committee in Biomedical Research of Mazandaran University of Medical Sciences (IR.MAZUMS.REC.1403.506).

## References

[1] Lindstrom M, DeCleene N, Dorsey H, Fuster V, Johnson CO, LeGrand KE (2022). Global burden of cardiovascular diseases and risks collaboration, 1990-2021. J Am Coll Cardiol.

[2] Twiner MJ, Hennessy J, Wein R, Levy PD (2022). Nitroglycerin use in the emergency department: current perspectives. Open Access Emerg Med.

[3] Sawan ZH, Soliman HM, Mostafa AE, Kamel RA (2023). A review on nitroglycerin: present and future. Egypt J Hosp Med.

[4] Miró Ò, Llorens P, Freund Y, Davison B, Takagi K, Herrero-Puente P (2021). Early intravenous nitroglycerin use in prehospital setting and in the emergency department to treat patients with acute heart failure: insights from the EAHFE Spanish registry. Int J Cardiol.

[5] Divakaran S, Loscalzo J (2017). The role of nitroglycerin and other nitrogen oxides in cardiovascular therapeutics. J Am Coll Cardiol.

[6] Kim KH, Kerndt CC, Adnan G, Schaller DJ. Nitroglycerin. In: StatPearls [Internet]. Treasure Island, FL: StatPearls Publishing; 2023.

[7] Hollas MA, Ben Aissa M, Lee SH, Gordon-Blake JM, Thatcher GRJ (2019). Pharmacological manipulation of cGMP and NO/cGMP in CNS drug discovery. Nitric Oxide.

[8] Pavasini R, Camici PG, Crea F, Danchin N, Fox K, Manolis AJ (2019). Anti-anginal drugs: systematic review and clinical implications. Int J Cardiol.

[9] Uğurlu YK, Enç N (2023). The effect of local cold compresses for nitroglycerin-induced headache: an observational pretest-posttest study. Nurs Crit Care.

[10] Yadollahi P, Taghizdeh Z, Ebadi A, Khormaei F (2019). Development and validation of the perception of labor pain questionnaire among Iranian women. Int J Community Based Nurs Midwifery.

[11] Moradi M, Jalili F, Basiri-Moghadam M, Basiri-Moghadam K (2022). Comparing the effect of aromatherapy with lavender and rose essential oils on the prevention of headache induced by infusion of nitroglycerin in cardiac care unit. J Isfahan Med Sch.

[12] Gonzalez A, Hyde E, Sangwan N, Gilbert JA, Viirre E, Knight R (2016). Migraines are correlated with higher levels of nitrate-, nitrite-, and nitric oxide-reducing oral microbes in the American gut project cohort. mSystems.

[13] Pellesi L (2023). The human NTG model of migraine in drug discovery and development. Expert Opin Drug Discov.

[14] Zarifnejad GH, Eshghi E, Mirhaghi A, Ghorbanzadeh H. The effect of aromatherapy with Lavender essential oil in treatment of headache due to nitroglycerine infusion in patient admitted to cardiac emergency department. Complementary Medicine Journal 2015:1248-57.

[15] Ong JJY, De Felice M (2018). Migraine treatment: current acute medications and their potential mechanisms of action. Neurotherapeutics.

[16] Tvedskov JF, Thomsen LL, Iversen HK, Gibson A, Wiliams P, Olesen J (2004). The prophylactic effect of valproate on glyceryltrinitrate induced migraine. Cephalalgia.

[17] Tfelt-Hansen P, Daugaard D, Lassen LH, Iversen HK, Olesen J (2009). Prednisolone reduces nitric oxide-induced migraine. Eur J Neurol.

[18] Fullerton T, Komorowski-Swiatek D, Forrest A, Gengo FM (1999). The pharmacodynamics of sumatriptan in nitroglycerin-induced headache. J Clin Pharmacol.

[19] Iversen HK, Olesen J (1996). Headache induced by a nitric oxide donor (nitroglycerin) responds to sumatriptan A human model for development of migraine drugs. Cephalalgia.

[20] Schmetterer L, Wolzt M, Krejcy K, Graselli U, Findl O, Eichler HG (1996). Cerebral and ocular hemodynamic effects of sumatriptan in the nitroglycerin headache model. Clin Pharmacol Ther.

[21] Mohammadi S, Yekefallah L (2023). Comparative study of the effect of aromatherapy with lavender and valerian essential oil on headache caused by nitroglycerin infusion, vital signs, and narcotic use in patients with coronary artery disease. Koomesh.

[22] Ezati-Soleiman R, Karampourian A, Khatiban M, Tapak L, Sayadi MH (2025). The effect of lavender aromatherapy and acetaminophen as preemptive on comfort and Nitroglycerin-induced headache in acute coronary syndrome. ARYA Atheroscler.

[23] Costa A, Pucci E, Antonaci F, Sances G, Granella F, Broich G (2000). The effect of intranasal cocaine and lidocaine on nitroglycerin-induced attacks in cluster headache. Cephalalgia.

[24] Tvedskov JF, Thomsen LL, Thomsen LL, Iversen HK, Williams P, Gibson A (2004). The effect of propranolol on glyceryltrinitrate-induced headache and arterial response. Cephalalgia.

[25] Lassen LH, Thomsen LL, Kruuse C, Iversen HK, Olesen J (1996). Histamine-1 receptor blockade does not prevent nitroglycerin induced migraine Support for the NO-hypothesis of migraine. Eur J Clin Pharmacol.

[26] Tvedskov JF, Tfelt-Hansen P, Petersen KA, Jensen LT, Olesen J (2010). CGRP receptor antagonist olcegepant (BIBN4096BS) does not prevent glyceryl trinitrate-induced migraine. Cephalalgia.

[27] Tvedskov JF, Iversen HK, Olesen J (2004). A double-blind study of SB-220453 (Tonerbasat) in the glyceryltrinitrate (GTN) model of migraine. Cephalalgia.

[28] Iversen HK (1992). N-acetylcysteine enhances nitroglycerin-induced headache and cranial arterial responses. Clin Pharmacol Ther.

[29] El Geziry A, Toble Y, Al Kadhi F, Pervaiz M, Al Nobani M. Non-pharmacological pain management. In: Shallik NA, ed. Pain Management in Special Circumstances. IntechOpen; 2018. doi: 10.5772/intechopen.79689.

[30] Moradi M, Mohammadzadeh H, Noori R, Basiri Moghadam K, Sadeghmoghadam L (2021). Effect of a sleep hygiene education program using telephone follow-up method on the sleep quality of the elderly. Iran J Ageing.

[31] Şapulu Alakan Y, Ünal E (2017). Pain assessment in intensive care nursing and nursing management. Hacettepe University Faculty of Health Sciences Journal.

[32] Fattahi M, Rejeh N, Heravi-Karimooi M, Tadrisi SD, Yazdani S, Saatchi K (2019). Effect of foot reflexology on headache due to nitroglycerin injection: a randomized controlled clinical trial. J Nurs Educ.

[33] Bagherzadi A, Emani R, Ghavami H, Khalkhali HR, Ebrahimi M (2021). Comparing the effect of heat and cold therapy on the intensity of nitrate induced migraine type headache in cardiac inpatients: a randomized controlled trial. Agri.

[34] Higgins JPT, Savović J, Page MJ, Elbers RG, Sterne JAC. Assessing risk of bias in a randomized trial. In: Cochrane Handbook for Systematic Reviews of Interventions. Wiley; 2019. p. 205-28. doi: 10.1002/9781119536604.ch8.

[35] Jadad AR, Moore RA, Carroll D, Jenkinson C, Reynolds DJ, Gavaghan DJ (1996). Assessing the quality of reports of randomized clinical trials: is blinding necessary?. Control Clin Trials.

[36] Imani N, Shams SA, Radfar M, Ghavami H, Khalkhali HR (2018). Effect of applying reflexology massage on nitroglycerin-induced migraine-type headache: a placebo-controlled clinical trial. Agri.

